# Supplementary material for: Molecular mass growth through ring expansion in polycyclic aromatic hydrocarbons via radical–radical reactions
Source: Nat Commun. 2019 Aug 15;10:3689. doi: 10.1038/s41467-019-11652-5 (PMC6695427; doi:10.1038/s41467-019-11652-5)
Supplement: Supplementary file 1 — Supplementary Information [file 41467_2019_11652_MOESM1_ESM.pdf]

Supplementary Information

Molecular Mass Growth Processes through Ring Expansion in Polycyclic Aromatic Hydrocarbons via Radical-Radical Reactions

Zhao et al.

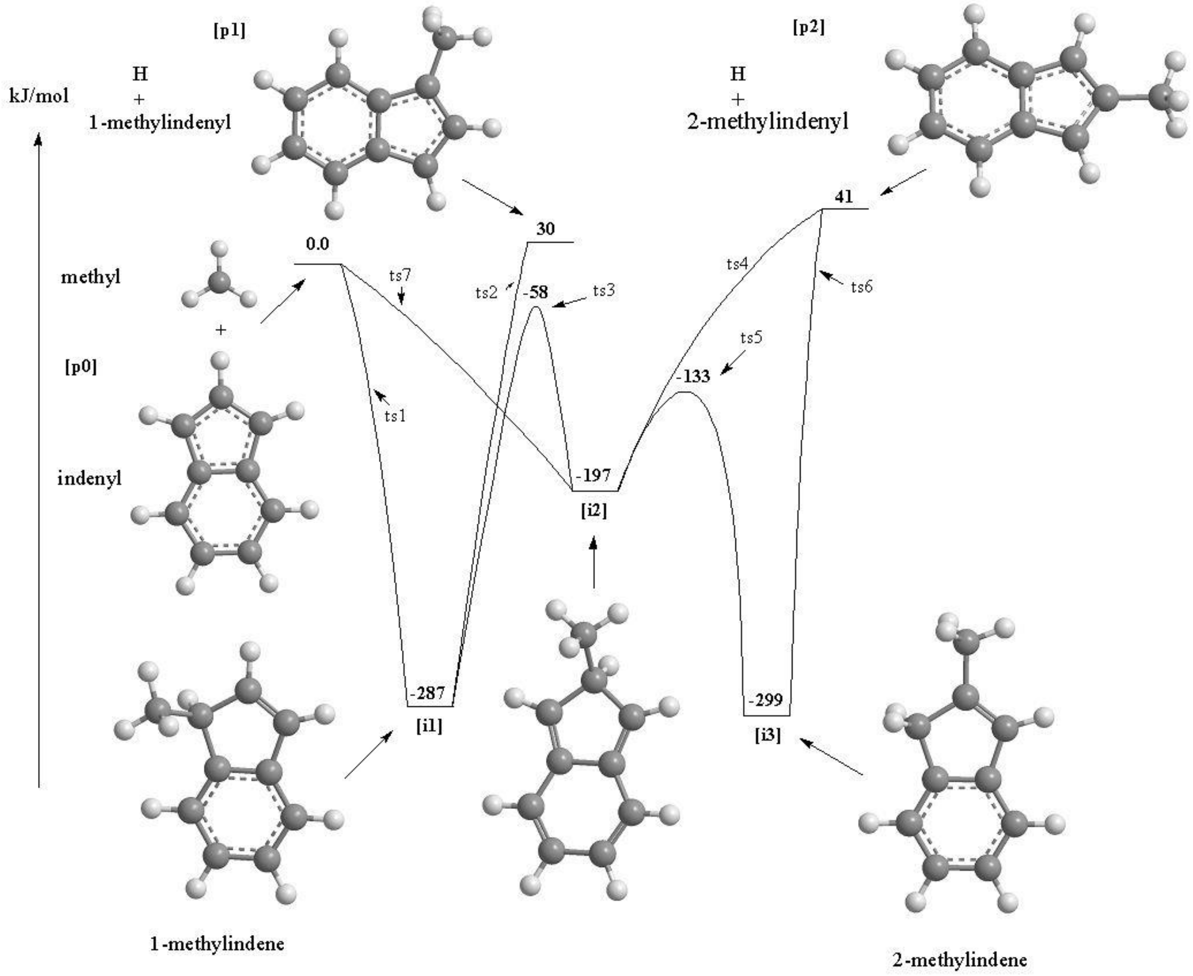

Supplementary Figure 1. Potential energy diagram for the indenyl + methyl reaction calculated at the CCSD(T)-F12/cc-pVTZ-f12//B3LYP/6-311G(d,p) + ZPE[B3LYP/6-311G(d,p)] level of theory. All relative energies are given in kJ/mol.

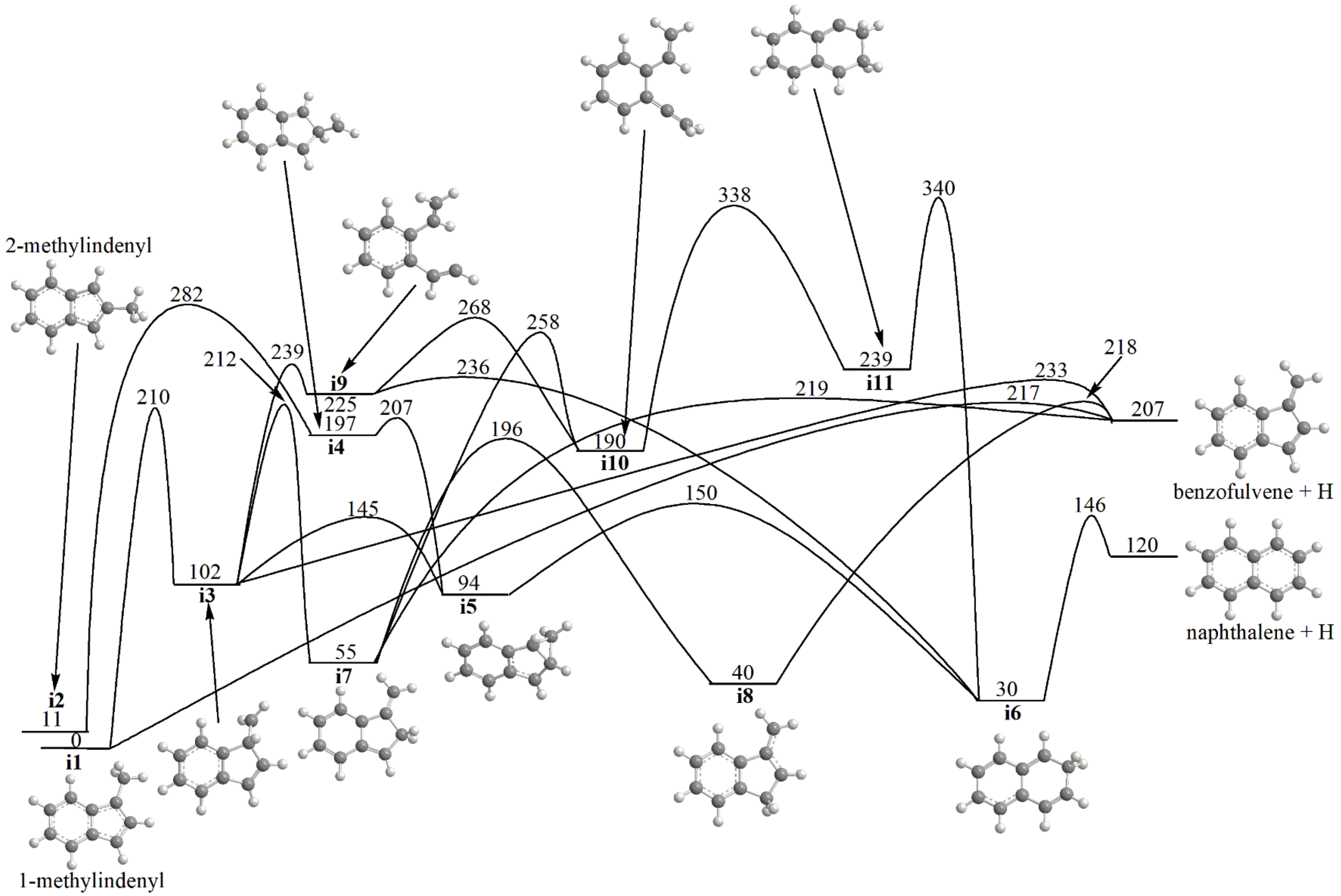

Supplementary Figure 2. Potential energy diagram for unimolecular decomposition of methylindenyl radicals calculated at the G3(CC,MP2)//B3LYP/6-311G(d,p) + ZPE[B3LYP/6-311G(d,p)] level of theory. All relative energies are given in kJ/mol. For more detail on this PES and temperature- and pressure-dependent rate constants see Ref. 22 in the main text.

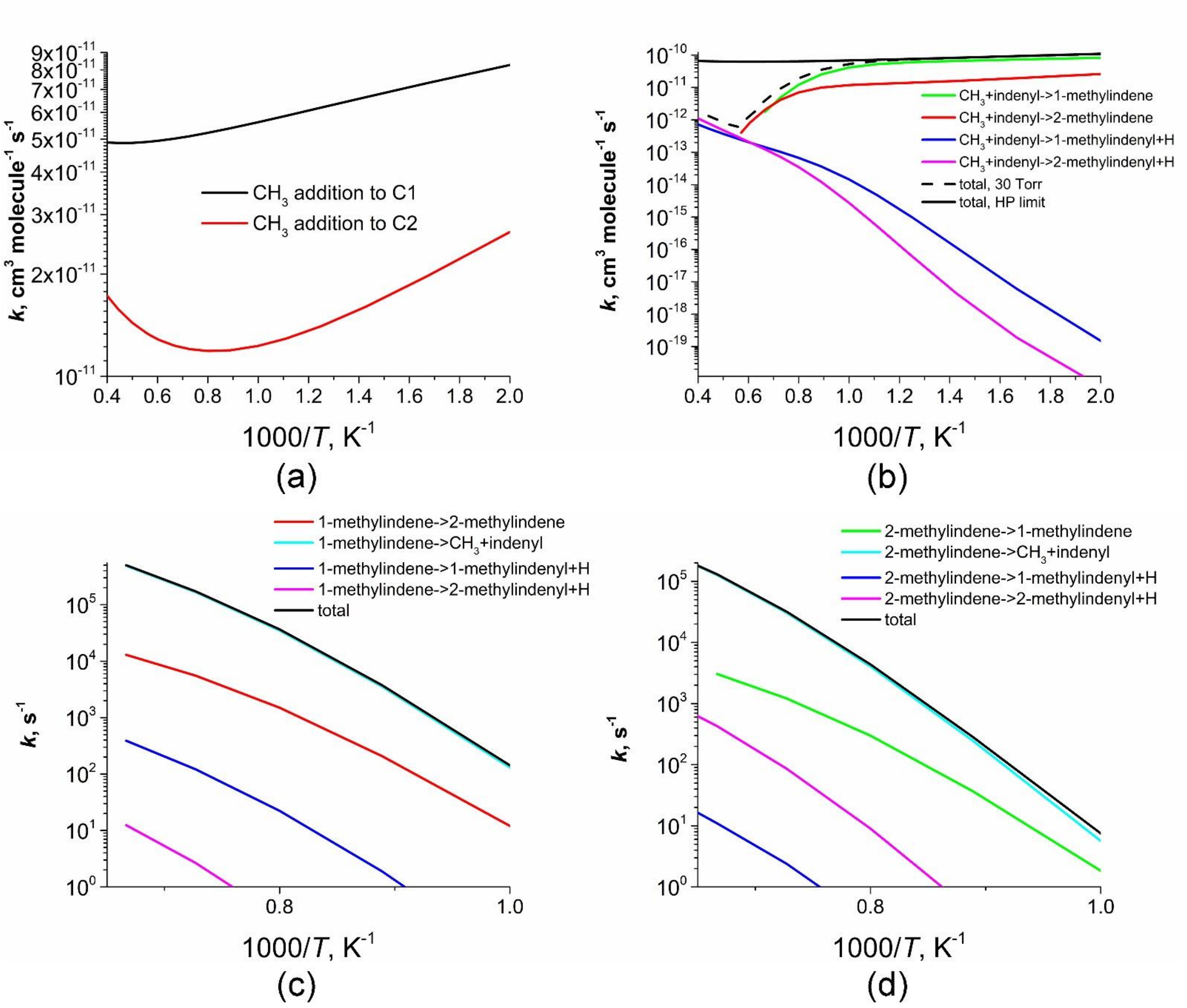
(a)
k, cm3 molecule-1 s-1
1000/T, K-1
CH3 addition to C1
CH3 addition to C2
(b)
CH3+indenyl->1-methylindene
CH3+indenyl->2-methylindene
CH3+indenyl->1-methylindenyl+H
CH3+indenyl->2-methylindenyl+H
total, 30 Torr
total, HP limit
(c)
k, s-1
1-methylindene->2-methylindene
1-methylindene->CH3+indenyl
1-methylindene->1-methylindenyl+H
1-methylindene->2-methylindenyl+H
total
(d)
2-methylindene->1-methylindene
2-methylindene->CH3+indenyl
2-methylindene->1-methylindenyl+H
2-methylindene->2-methylindenyl+H
total

Supplementary Figure 3.

(a) High-pressure limit rate constants for CH_3 addition to C1/C3 vs. C2 in indenyl. In the entrance channel, the addition to C1/C3 is favored, by a factor of ~4 at *T* about 1400 K, i.e., in the reactor's temperature range.

(b) Total and individual channel rate constants at 30 Torr (the typical pressure inside the reactor). Fitted modified Arrhenius expressions for all relevant reactions in the indenyl + CH_3 system calculated at various pressures up to 100 atm (for combustion applications) are presented in Table S1. There is a competition between collisional stabilization of 1- and 2-methylindene and the formation of bimolecular 1-/2-methylindenyl + H products. The calculated product branching ratios at 30 Torr are shown in Table S2. In the 1375-1500 K temperature range, collisionally stabilized 1- and 2-methylindene are predicted to be the predominant products with nearly equal yields, whereas the contribution of 1-/2-methylindenyl + H via well skipping pathways is only few percent.

(c) Unimolecular rate constants for isomerization/dissociation of 1-methylindene at 30 Torr.

(d) Unimolecular rate constants for isomerization/dissociation of 2-methylindene at 30 Torr.

Both methylindenes preferably decompose back to the reactants and the second most probable channel for each is isomerization to one another followed by H loss to 1- and 2-methylindenyl radicals, respectively. According to the calculated rate constants, 2-methylindene is more stable with the equilibrium constant for 1-methylindene $\rightarrow$ 2-methylindene being ~4.5 in the microreactor's temperature range. Therefore, 2-methylindene should be significantly more abundant on the exit from the reactor in agreement with the PIE fit for *m/z* = 131.

Source data are provided as a Source Data file.

Supplementary Table 1. Rate constant parameterization using modified Arrhenius expressions $k = A_1{*}T^{\alpha_1}{*}\exp(-E_1/T) + A_2{*}T^{\alpha_2}{*}\exp(-E_2/T)$. Pre-exponential factors A are in cm^3 $molecule^{-1}$ s^{-1} for bimolecular reactions and in s^{-1} for unimolecular reactions, T in K.

**indenyl + methyl →**	***p***	***A*₁**	$\boldsymbol{\alpha_1}$	***E*₁**	***A*₂**	$\boldsymbol{\alpha_2}$	***E*₂**	***T*-range, K**
→ C1/C3	HP	1.3276E-09	-0.49466	-142.31	1.2287E-12	0.42337	1359.1	500-2500
→ C2	HP	1.4856E-13	0.44791	-1200.3	1.6922E-15	1.1692	1347.9	500-2500
**indenyl + methyl →**	***p***	***A*₁**	$\boldsymbol{\alpha_1}$	***E*₁**	***A*₂**	$\boldsymbol{\alpha_2}$	***E*₂**	***T*-range, K**
→ 1-methylindene	30Torr	-2.09E+76	-26.327	17489	8.34E+54	-19.763	12988	500-1500
	1atm	1.97E-12	0.41028	-588.75	-2.78E+25	-9.653	17680	500-1750
	10atm	2.05E-16	1.6227	-1423.2	-4.48E+10	-5.3914	13296	500-1800
	100atm	2.42E+53	-17.95	23881	41667	-4.6437	2523.8	500-2000
→ 2-methylindene	30Torr	2.85E+89	-29.108	30323	5.20E+22	-10.609	5400.4	500-1750
	1atm	9.73E+71	-23.607	28565	9.23E+11	-7.2004	3632.3	500-2000
	10atm	8.23E+67	-22.082	30750	1.91E+08	-5.9183	3613	500-2250
	100atm	5.88E+34	-12.738	18733	1.56E+07	-5.4336	4716	500-2500
→ 1-methylindenyl + H	30Torr	1.21E+16	-7.5678	16791	0.15850+104	-29.706	84154	500-2500
	1atm	1.22E+20	-8.4071	21679	0.39030+140	-47.71	35778	500-2500
	10atm	8.30E+17	-7.6401	23488				700-2500
	100atm	4.52E-22	3.0339	10497	7.74E+32	-11.55	33620	500-2500
→ 2-methylindenyl + H	30Torr	4.24E+11	-5.9831	19105				600-2500
	1atm	1.22E-28	4.9539	6759.3	4.02E+36	-12.735	32143	500-2500
	10atm	1.15E+39	-13.192	36951	2.49E-26	4.3031	7586.7	500-2500
	100atm	2.21E+15	-6.6829	28484	1.61E-23	3.5775	9232.5	500-2500
**1-methyindene →**	***p***	***A*₁**	$\boldsymbol{\alpha_1}$	***E*₁**	***A*₂**	$\boldsymbol{\alpha_2}$	***E*₂**	***T*-range, K**
→ 2-methylindene	30Torr	0.13617+120	-30.879	58529				1000-1500

	1atm	0.81637+116	-29.309	62662	2.66E+74	-18.404	42241	800-1750
	10atm	-3.11E+56	-12.839	36689	1.11E+46	-9.7049	34166	800-1800
	100atm	2.98E+75	-17.472	51788	4.46E+36	-6.9006	35180	500-2000
→indenyl + methyl	30Torr	0.45451+109	-27.153	57707				800-1500
	1atm	6.79E+22	-1.6881	35695	-1.29E+64	-12.877	55919	500-1750
	10atm	8.26E+20	-1.1193	35240	-1.57E+75	-15.699	63794	500-1800
	100atm	2.61E+80	-17.954	57048	1.75E+38	-6.4844	38540	500-2000
→ 1-methylindenyl + H	30Torr	0.14432+129	-33.403	67269				900-1500
	1atm	0.42379+107	-26.563	64346				900-1750
	10atm	1.24E+12	1.0987	37683	-2.83E+52	-9.7794	58301	500-1800
	100atm	6.05E+09	1.8024	37196	-8.12E+33	-4.5608	51746	500-2000
→ 2-methylindenyl + H	30Torr	0.30003+145	-37.841	80110				1000-1500
	1atm	0.37180+119	-29.783	77225				1000-1750
	10atm	5.65E-04	5.3376	39628	-6.89E+13	0.86649	50239	500-1800
	100atm	3.75E+65	-14.42	62486	1.28E+28	-4.3178	46389	500-2000
**2-methyindene →**	***p***	A_1	α_1	E_1	A_2	α_2	E_2	***T*-range, K**
→ 1-methylindene	30Torr	0.31098+121	-31.333	60399				1000-1500
	1atm	0.58750+124	-31.56	67276	5.20E+77	-19.374	44883	800-1750
	10atm	0.21644+101	-24.772	60424	6.41E+39	-8.0221	35916	500-1800
	100atm	1.91E+08	1.8675	31362	-4.33E+20	-1.4118	38480	500-2000
→indenyl + methyl	30Torr	0.97281+113	-28.108	64286				1000-1750
	1atm	2.36E+98	-23.473	63235	3.93E+43	-8.4978	40662	500-2000
	10atm	7.49E+91	-21.318	63973	5.45E+40	-7.4667	40817	500-2250
	100atm	3.90E+63	-13.328	53762	5.00E+40	-7.325	42062	500-2500
→ 1-methylindenyl + H	30Torr	0.39384+133	-34.474	76064				1000-1750
	1atm	0.15172+127	-31.885	80915				1000-2000
	10atm	0.44243+112	-27.324	80934				1000-2250

	100atm	1.18E+90	-20.965	77544				1000-2500
→ 2-methylindenyl + H	30Torr	0.33759+126	-32.046	72923				1000-1750
	1atm	0.88645+106	-25.819	71035	1.28E+47	-9.718	47404	500-2000
	10atm	9.64E+90	-21.191	69088	7.79E+38	-7.103	46109	500-2250
	100atm	1.95E+67	-14.316	62473	9.91E+31	-4.9091	45212	500-2500

Supplementary Table 2. The calculated branching ratios (%) for the main channels of indenyl + methyl reaction.

***p* = 30 Torr**				
	**1-methylindene**	**2-methylindene**	**1-methylindenyl +H**	**2-methylindenyl +H**
500 K	76.1	23.9	0.0	0.0
600 K	79.0	21.0	0.0	0.0
700 K	80.6	19.4	0.0	0.0
800 K	81.2	18.8	0.0	0.0
900 K	80.4	19.6	0.0	0.0
1000 K	77.8	22.2	0.0	0.0
1125 K	71.7	28.2	0.1	0.0
1250 K	62.9	36.5	0.3	0.2
1375 K	53.1	45.0	1.1	0.8
1500 K	43.1	50.5	3.5	3.0
1750 K		44.1	26.4	29.2
2000 K			43.5	56.5
2250 K			41.0	59.0
2500 K			39.4	60.6

Supplementary Note 1. Synthetic Procedure:

1-Bromoindene was synthesized using the procedure described in detail in our previous publication.[1]

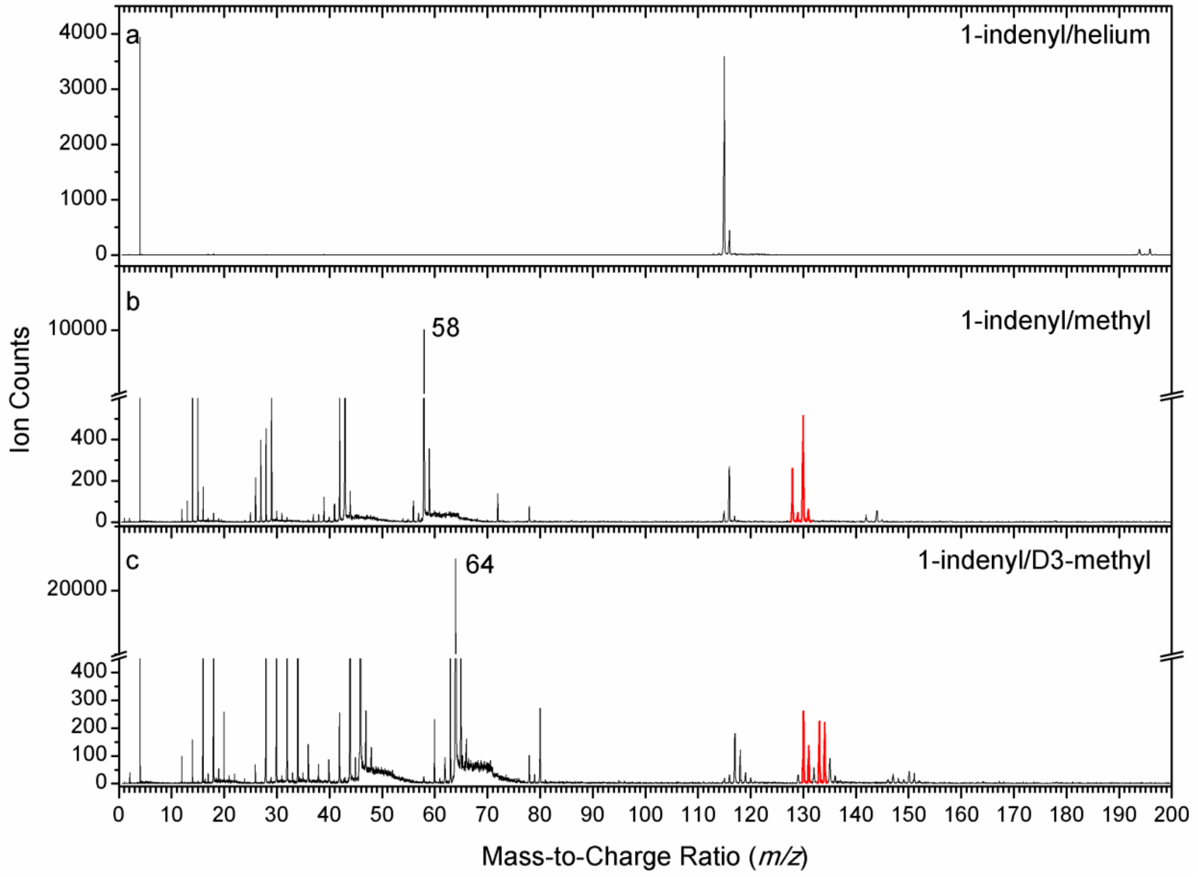

Supplementary Figure 4. Complete mass spectra recorded at a photon energy of 9.50 eV; the source temperature was 1425 ± 10 K. (a) 1-indenyl/helium, (b) 1-indenyl/ methyl, (c) 1-indenyl/D3-methyl. Source data are provided as a Source Data file.

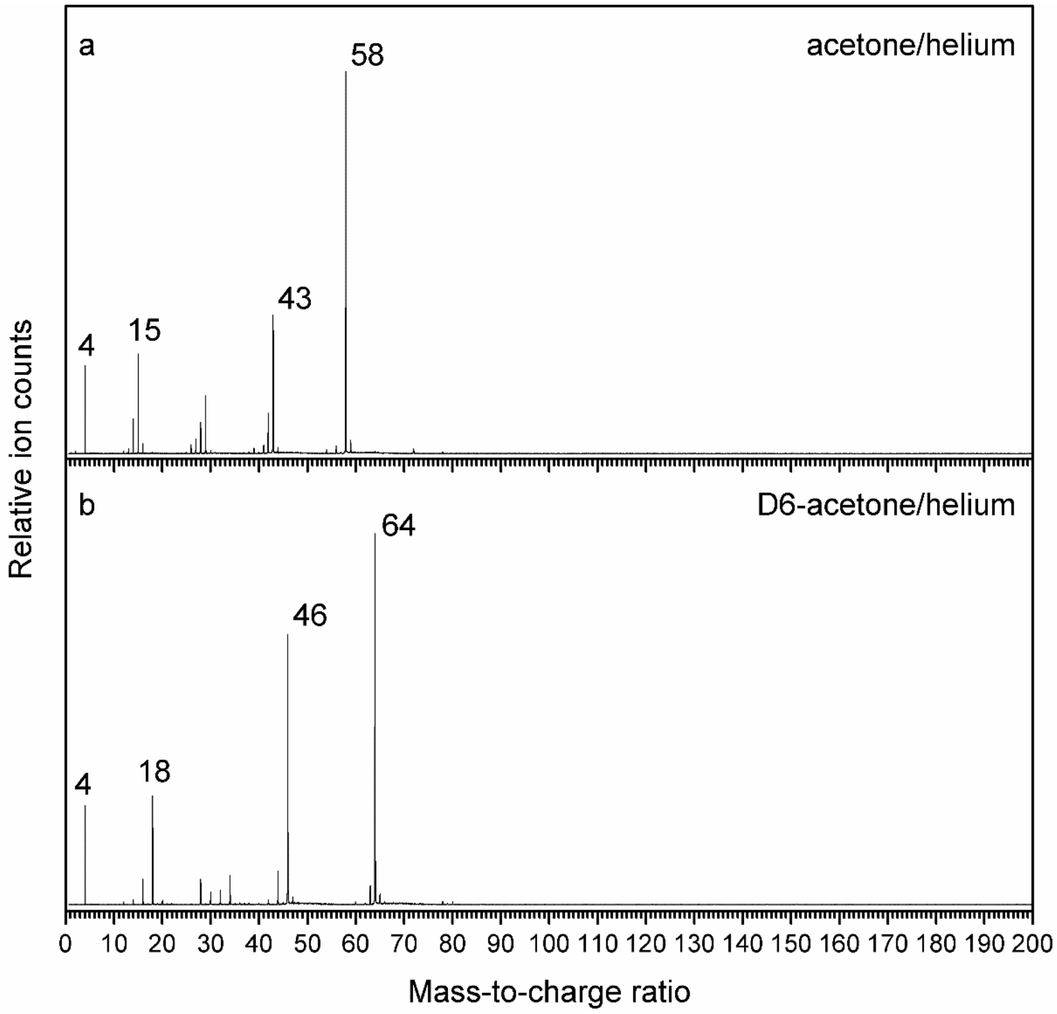

Supplementary Figure 5. Mass spectra recorded at a photon energy of 9.50 eV; the source temperature was 1425 ± 10 K. (a) acetone/helium, (b) D6-acetone/helium.

In both acetone – helium and D6-acetone – helium systems, helium was observed at *m/z* = 4, with a photoionization energy of 24.6 eV,[2] due to the high harmonics of VUV light and the high concentration of helium. In the acetone – helium system, another three strong peaks at *m/z* = 15, 43 and 58 are detected. Signal at *m/z* = 58 is attributed to acetone (CH_3COCH_3). With the C-C bond scission, acetone is decomposed to acetyl ($CH_3CO^•$) and methyl ($CH_3^•$) radicals, which are at *m/z* = 15 and 43. The species at *m/z* = 42 should be ketene (CH_2CO), the H-loss product of acetyl radical.

In the D6-acetone – helium system, strong signal at *m/z* = 18, 46 and 64 is observed. Similar as that in the acetone – helium system, these three peaks are attributed to D3-methyl ($CD_3^•$), D3-acetyl ($CD_3CO^•$) and D6-acetone (CD_3COCD_3), respectively, with the previous two generated from the C-C scission of D6-acetone. Analogously, D2-ketene (CD_2CO, *m/z* = 44) is produced via D-loss of D3-acetyl. For other small signal masses, as the goal in this work is to investigate the indenyl – methyl reaction, they are not discussed in detail here.

Source data are provided as a Source Data file.

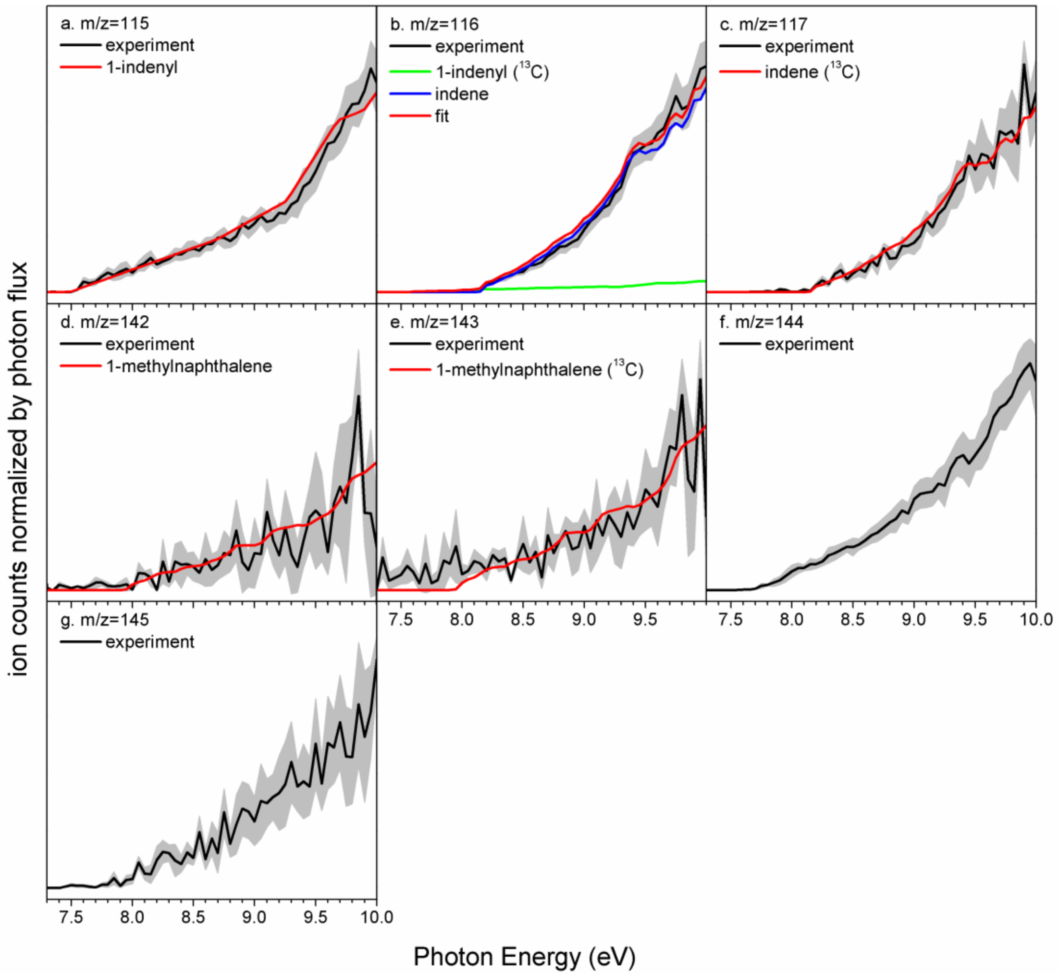

Supplementary Figure 6: PIE curves of additional ion counts detected in the 1-indenyl – methyl system. The detected signals include: (a) *m/z* = 115, (b) *m/z* = 116, (c) *m/z* = 117, (d) *m/z* = 142, (e) *m/z* = 143, (f) *m/z* = 144 and (g) *m/z* = 145. Black: experimental PIE curves; blue/green/red: reference PIE curves. In case of multiple contributions to one PIE curve, the red line resembles the overall fit. The error bars consist of two parts: ±10% based on the accuracy of the photodiode and a 1 σ error of the PIE curve averaged over the individual scans.

Source data are provided as a Source Data file.

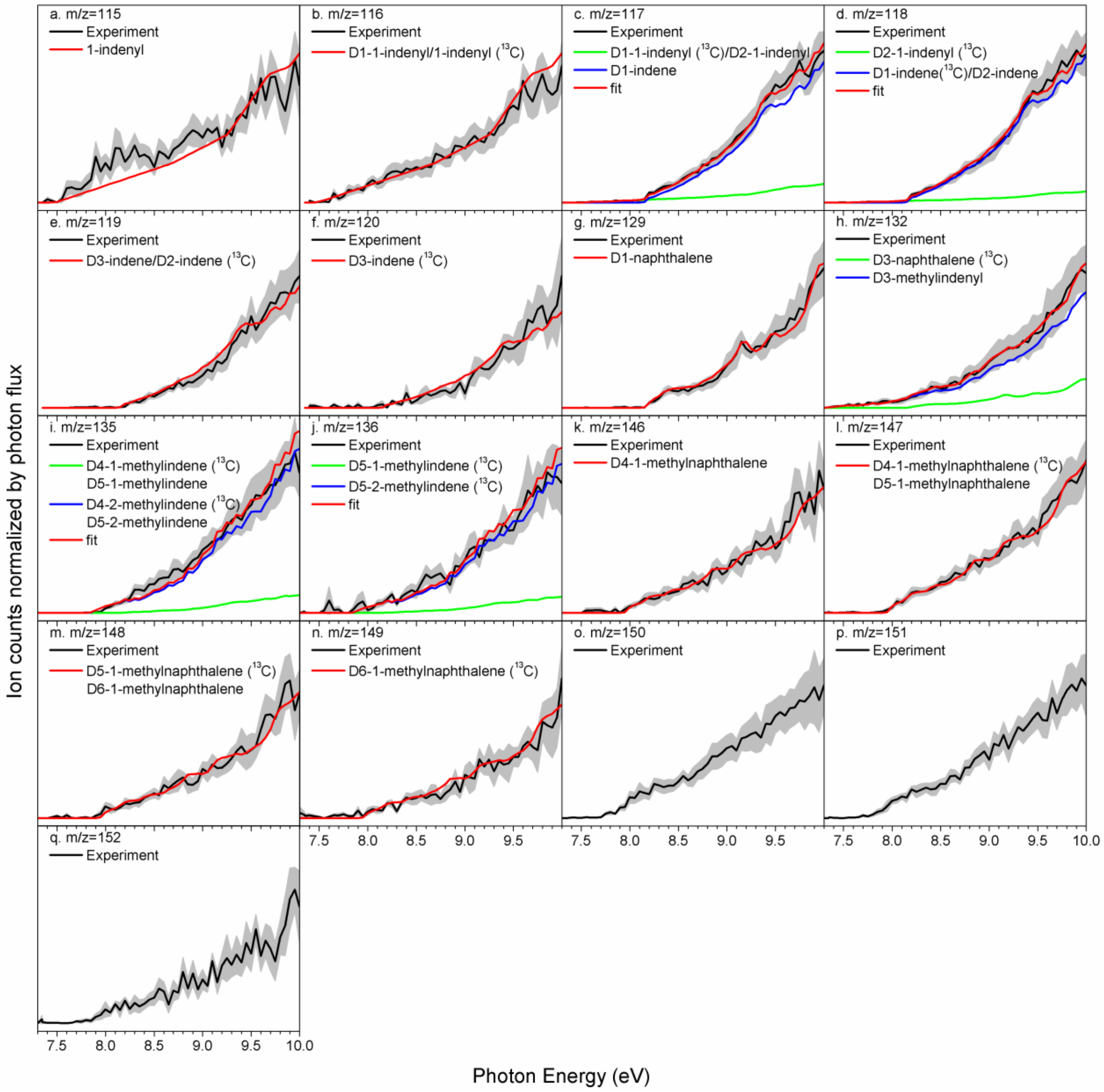

Supplementary Figure 7: PIE curves of additional ion counts detected in the 1-indenyl – D3-methyl system. PIE fits for signals at (a) *m/z* = 115, (b) *m/z* = 116, (c) *m/z* = 117, (d) *m/z* = 118, (e) *m/z* = 119, (f) *m/z* = 120, (g) *m/z* = 129, (h) *m/z* = 132, (i) *m/z* = 135, (j) *m/z* = 136, (k) *m/z* = 146, (l) *m/z* = 147, (m) *m/z* = 148, (n) *m/z* = 149, (o) *m/z* = 150, (p) *m/z* = 151 and (q) *m/z* = 152 are presented. Black: experimental PIE curves; blue/green/red: reference PIE curves. In case of multiple contributions to one PIE curve, the red line resembles the overall fit. The error bars consist of two parts: ±10% based on the accuracy of the photodiode and a 1 σ error of the PIE curve averaged over the individual scans.

Source data are provided as a Source Data file.

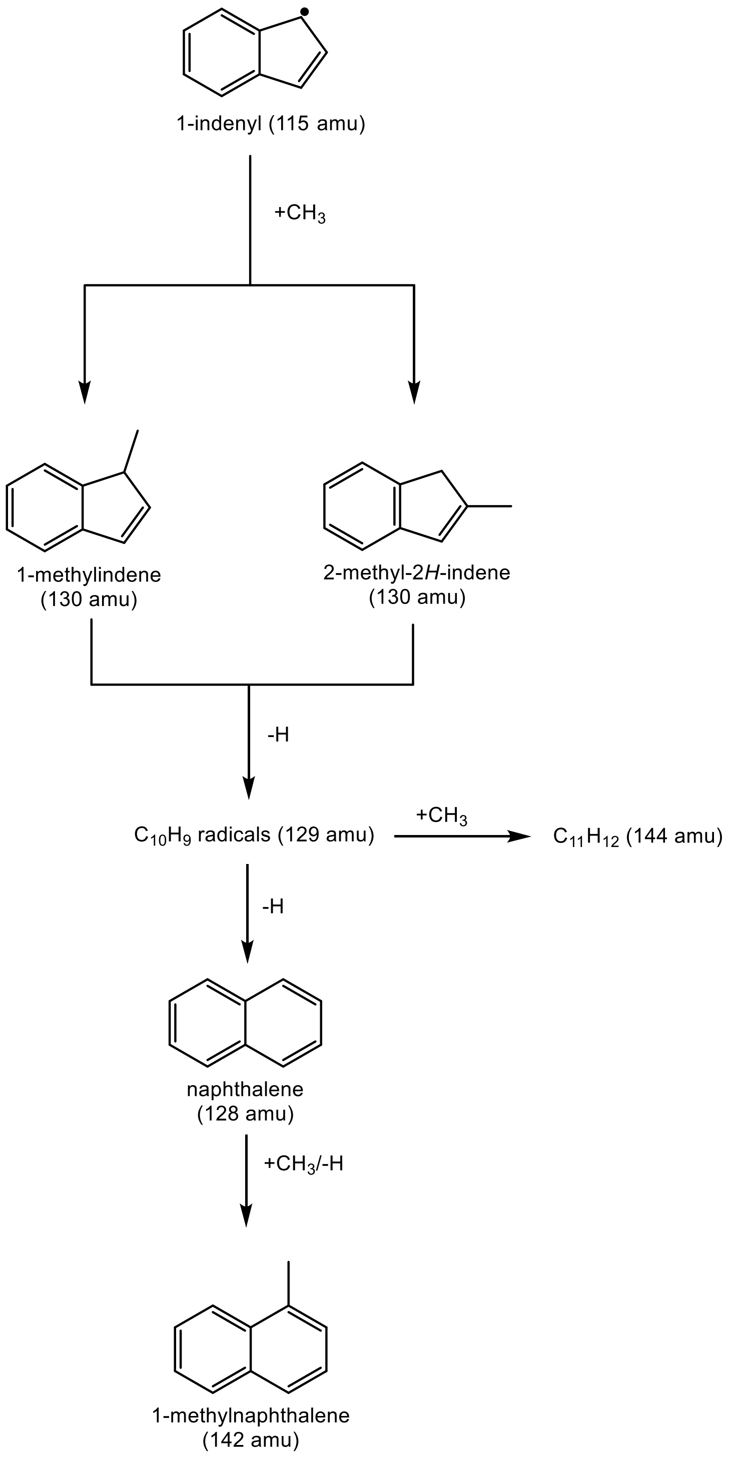

Supplementary Figure 8: Schematic reaction pathways in the 1-indenyl – methyl system to account for ion counts from *m/z* = 142 to 145.

In the 1-indenyl - methyl system, ion signal from *m/z* = 142 to 145 was also observed. The analysis of the PIE curves reveals that *m/z* = 142 ($C_{11}H_{10}^+$) and 143 ($^{13}CC_{10}H_{10}^+$) are attributed to 1-methylnaphthalene and ^{13}C-1-methylnaphthalene, respectively (Supplementary

Figs. 6(d) and (e)). This species might be generated via the reaction of naphthalene with a methyl radical followed by hydrogen atom loss.[3] Considering the ion counts at *m/z* = 144 ($C_{11}H_{12}^+$) and 145 ($^{13}CC_{10}H_{12}^+$), the PIE curves overlap after scaling indicating that *m/z* = 145 is the ^{13}C counterpart of *m/z* = 144 (Supplementary Figs. 6(f) and (g)). Considering the molecular weight, this species might be produced via the reaction of methylindene ($C_{10}H_{10}$, *m/z* = 130) and methyl ($CH_3^{\bullet}$, *m/z* = 15) followed by atomic hydrogen loss. However, due to the lack of any reference PIE data for dimethylated indene isomers, *m/z* = 144 and 145 remain unidentified.

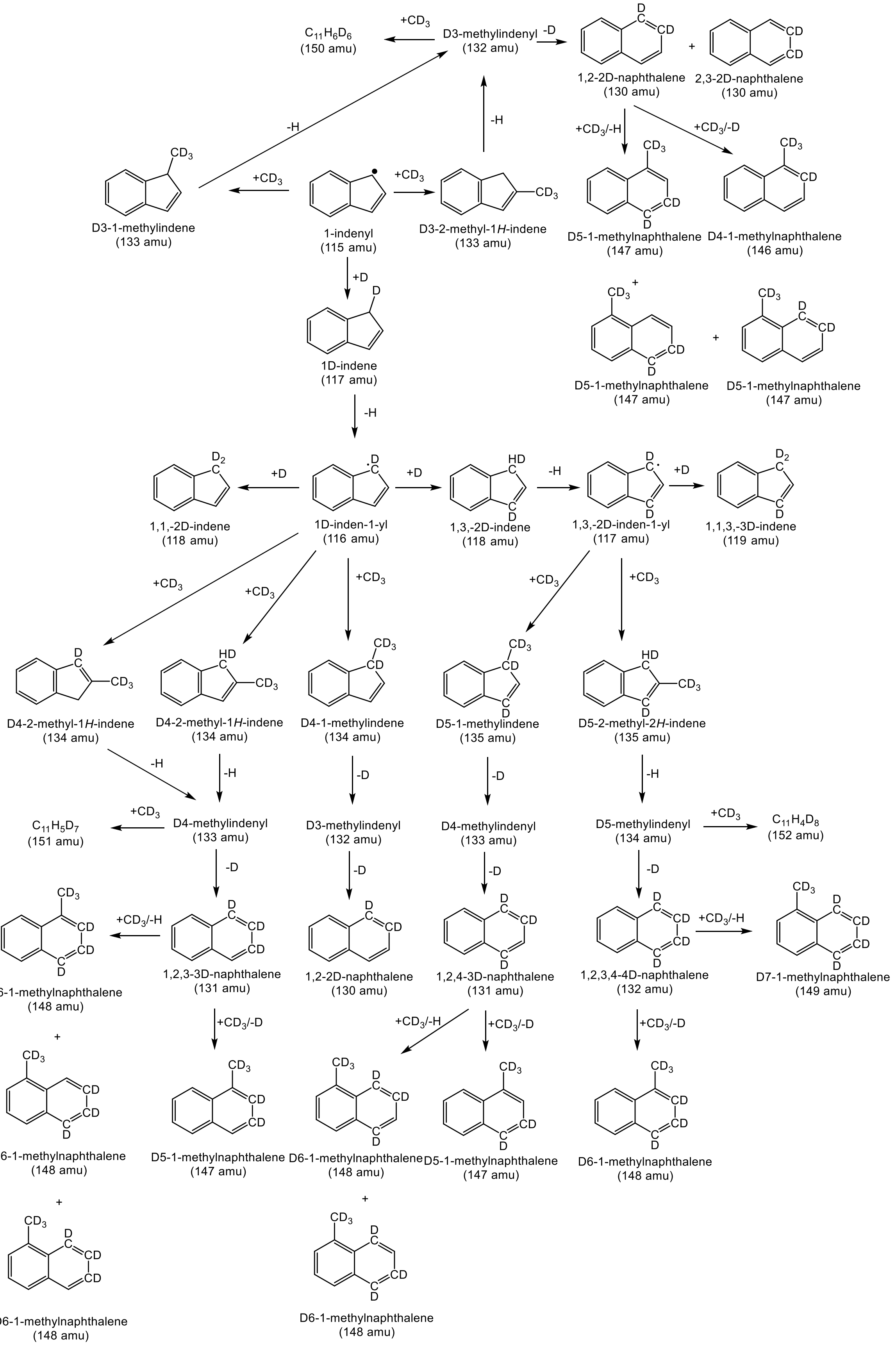

Supplementary Figure 9: Schematic reaction pathways in the 1-indenyl – D3-methyl system.

The calculated branching ratios of benzofulvene versus naphthalene at the conditions of our reactor are nearly equal to 4 to 1. Therefore, we expected the bimolecular mechanism, i.e. deuterium (D) loss from methylindenyl to eventually form benzofulvene followed by H-assisted isomerization of the latter to naphthalene, to be the most favorable mechanism. This mechanism is consistent with the isotope-labeling result if benzofulvene re-reacts with D and isomerizes to naphthalene through such D-assisted isomerization. However, it is not consistent if benzofulvene reacts with H rather than with D - then the reaction would produce naphthalene with only one D incorporated (*m/z* = 129). The concentrations of hydrogen and deuterium are expected to be roughly equal because the dissociation of 1- or 2-methylindene produces hydrogen and the dissociation of D3-methylindenyl radicals produces deuterium. Therefore, technically these calculations show that 1/5 of naphthalene is produced via the unimolecular mechanism containing two deuterium atoms; 2/5 of naphthalene is likely produced via the bimolecular mechanism via a deuterium-assisted isomerization of benzofulvene containing also two deuterium atoms; finally, 2/5 of naphthalene are formed via a hydrogen-assisted isomerization of benzofulvene and contain only one deuterium. Therefore, qualitatively speaking, the intensity of the ion counts at *m/z* = 130 are expected to be higher than at *m/z* = 129, which agrees with our experimental findings. Further H/D isotope scrambling involving naphthalene itself, like H + $C_{10}H_6D_2$ (naphthalene) $\leftrightarrows$ D + $C_{10}H_7D_1$ (naphthalene) is favorable in reverse direction because a C-D bond is stronger than a C-H bond due to the effect of ZPE and thus, the H/D isotope scrambling will additionally boost the *m/z* = 130 peak over *m/z* = 129.

Recall that in the 1-indenyl – methyl system, signal is detected from *m/z* = 115 to 117 (1-indenyl, ^{13}C-1-indenyl/indene and ^{13}C-indene); in the 1-indenyl – D3-methyl system (Supplementary Fig. 7), six distinct peaks were observable from *m/z* = 115 to 120. The fits of the PIE graphs indicate that – as detailed below – these ion counts can be connected with the formation of (deuterium substituted) 1-indenyl radicals and indenes including 1-indenyl, D1- and D2-1-indenyls, D1-, D2- and D3-indenes, as well as their ^{13}C-substitued counterparts (Supplementary Figs. 7(a)-(f)). The 1-indenyl radical ($C_9H_7^{\bullet}$, *m/z* = 115, Supplementary Fig. 7(a)) is produced from the pyrolysis of the precursor 1-bromoindene via homolytic carbon – bromine bond (C-Br) scission. The deuterated 1-indenyl radicals might be generated from 1-indenyl radical via two successive D-addition followed by H-loss. For instance, the D-addition to 1-indenyl generates 1D-indene (C_9H_7D, *m/z* = 117, Supplementary Fig. 7(c)), followed by H-loss via either bond scission or H/D-exchange leading to the formation of 1D-

inden-1-yl ($C_9H_6D^•$, *m/z* = 116, Supplementary Fig. 7(b)). Furthermore, combination of 1D-inden-1-yl and D atom yields D2-indenes including 1,1-2D-indene and 1,3-2D-indene ($C_9H_6D_2$, *m/z* = 118, Supplementary Figs. 7(d) and S9).[4] Analogously, 2D-indenyl radicals ($C_9H_5D_2^•$, *m/z* = 117, Supplementary Fig. 7(c)) and 3D-indene ($C_9H_5D_3$, *m/z* =119, Supplementary Fig. 7(e)) are produced in the subsequent reactions. The signal at *m/z* = 120 is attributed to the ^{13}C 3D-indene ($^{13}CC_8H_5D_3$, Supplementary Fig. 7(f)).

The reactions of the 1-indenyl radicals with D3-methyl result in D2-, D3-, D4-naphthalenes ($C_{10}H_6D_2$, *m/z* = 130, Fig. 3(e); $^{13}CC_9H_6D_2$/$C_{10}H_5D_3$, *m/z* = 131, Fig. 3(f); $^{13}CC_9H_5D_3$/ $C_{10}H_4D_4$, *m/z* = 132, Supplementary Fig. 7(h)) and D3-, D4-, D5-substituted 1- and 2-methylindenes ($C_{10}H_7D_3$, *m/z* = 133, Fig. 3(g); $^{13}CC_9H_7D_3$/$C_{10}H_6D_4$, *m/z* = 134, Fig. 3(h); $^{13}CC_9H_6D_4$/$C_{10}H_5D_5$, *m/z* = 135, Supplementary Fig. 7(i)). Signal at *m/z* = 136 is related to ^{13}C-D5-1-methylindene and ^{13}C-D5-2-methylindene ($^{13}CC_9H_5D_5$, Supplementary Fig. 7(j)). Note that D3-methylindenyl radical also contributes to the signal at *m/z* = 132 (Fig. S7(h)). Please also note the signal at *m/z* = 129 (Supplementary Fig. 7(g)) is attributed to D1-naphthalene. The reaction of 1-indenyl plus deuterium forming D1-indene followed by decomposition to D1-indenyl plus atomic hydrogen is exoergic because the C-H bond is weaker than the C-D bond due to the effect of zero-point vibrational energy. The relatively strong signals of the deuterated indenyl radicals (Supplementary Figures 4c and 7) also suggest the existence of D1-1-indenyl radical, one of the precursors of D4-methylindene. These bimolecular reactions show the potential formation processes for deuterated indenyl radicals, and they do not go against the fact that naphthalene is formed via a 1-indenyl - methyl recombination followed by H-loss steps. Just like the formation of D3-methylindene via the reaction of 1-indenyl with D3-methyl, D4-methylindene is produced via the reaction of D1-1-indenyl with D3-methyl. In conclusion, the major source of paramount 133 and 134 peaks may not be mainly from bimolecular reactions, but they should originate from different indenyl precursors.

Signal of *m/z* = 146 to 149 (Supplementary Figs. 7(k)-(n)) is attributed to D4-, D5- and D6-1-methylnaphthalenes and their ^{13}C counterparts ($C_{11}H_6D_4$, *m/z* = 146, Supplementary Fig. 7(k); $^{13}CC_{10}H_6D_4$/$C_{11}H_5D_5$, *m/z* = 147, Supplementary Fig. 7(l); $^{13}CC_{10}H_5D_5$/$C_{11}H_4D_6$, *m/z* = 148, Supplementary Fig. 7(m); $^{13}CC_{10}H_4D_6$, *m/z* = 149, Supplementary Fig. 7(n)). These deuterated methylnaphthalenes are proposed to be generated via the corresponding deuterated naphthalene with D3-methyl followed by H- or D-loss.[3]

Analogous to the 1-indenyl – methyl system, signal at *m/z* = 150 to 152 (Supplementary Figs. 7(o)-(q)) is attributed to products via the reaction of D3-, D4-, D5-methylindenes ($C_{10}H_7D_3$, *m/z* = 133; $C_{10}H_6D_4$, *m/z* =134; $C_{10}H_5D_5$, *m/z* = 135) and D3-methyl after H- or D-loss. Due to the lack of reference PIE data, species at *m/z* = 150 to 152 remain unidentified. With deuteration in different carbon positions in the 1-indenyl radical, subsequent reactions lead to a variety of deuterated products.

Please note that we cannot differentiate between ^{13}C-D3-methylindene and D4-methylindene. Based on the isotope ratio and the experimental findings, this would suggest that we are making copious amounts of D4-methylindene, which means that a deuterium atom is also substituting a hydrogen atom in the ring as described in Supplementary Figure 9 in the Supplementary Material. Here, the reaction of indenyl plus deuterium forming D1-indene followed by decomposition to D1-indenyl plus atomic hydrogen is exoergic because the C-H bond is weaker than the C-D bond due to the effect of zero-point vibrational energy. This process is followed by the reaction of D1-indenyl plus D3-methyl to give D4-methylindene (*m/z* = 134).

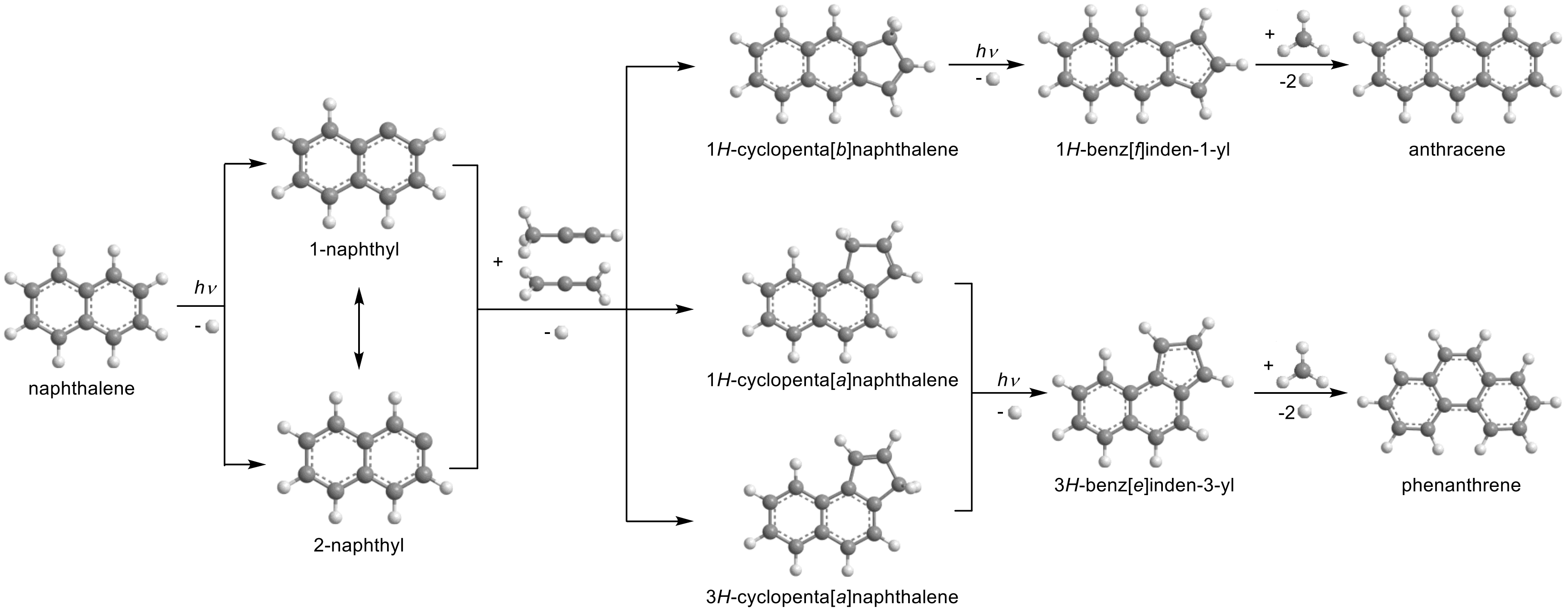

Supplementary Figure 10: Schematic hydrogen abstraction - methylacetylene/allene addition pathways leading to distinct cyclopentanaphthalenes isomers. Successive radical-radical mechanisms could form anthracene and phenanthrene. This pathway is presented schematically for interstellar environments (photon induced hydrogen loss); in combustion systems, the hydrogen atom can be lost via hydrogen abstraction by hydrogen atoms ubiquitous in flames.

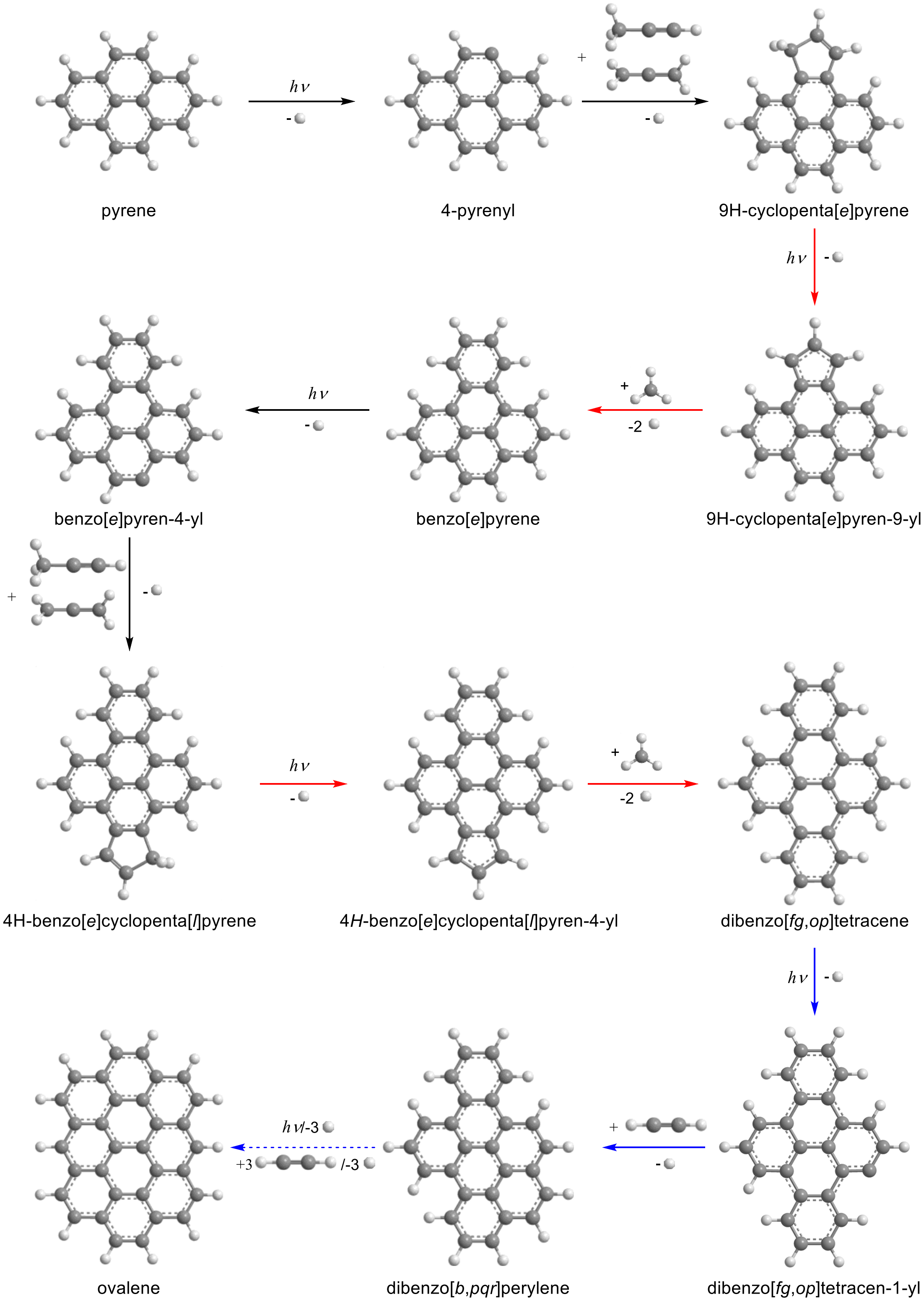

Supplementary Figure 11: Schematic representation of the complementary nature of the novel radical-radical (red) and the hydrogen abstraction-acetylene addition (HACA) mechanisms (blue) leading to planar PAHs. This pathway is presented schematically for extraterrestrial environments (photon induced hydrogen loss); in combustion systems, the hydrogen atom can be abstracted by a hydrogen atom ubiquitous in flames.

Pyrene ($C_{16}H_{10}$) can be photolyzed by interstellar UV photons leading to the formation of the 4-pyrenyl radical ($C_{16}H_9^•$), which reacts with allene/methylacetylene to yield 9H-cyclopenta(e)pyrene ($C_{19}H_{12}$). The latter can also be photolyzed yielding 9H-cyclopenta(e)pyren-9-yl ($C_{19}H_{11}^•$), which then can react with the methyl radical leading to benzo[*e*]pyrene ($C_{20}H_{12}$). Similarly, benzo[*e*]pyrene is proposed to follow the sequence of photon induced hydrogen loss to benzo[*e*]pyren-4-yl, allene/methylacetylene addition to 4H-benzo[*e*]cyclopenta[*l*]pyrene, photon induced hydrogen loss to 4H-benzo[*e*]cyclopenta [*l*]pyren-4-yl, and finally the reaction with a methyl radical generating dibenzo[*fg,op*] tetracene ($C_{24}H_{14}$). Dibenzo[*fg,op*]tetracene undergoes the successive steps of photon induced hydrogen loss and hydrogen abstraction-acetylene addition (HACA) mechanisms leading eventually to the formation of a more complex two-dimension PAH ovalene ($C_{32}H_{14}$).

Supplementary Note 2: Cartesian coordinates, vibrational frequencies, relative energies and hindered rotor potentials for various species in the indenyl + CH_3 reaction in the form of MESS input file.

```
!-------c10h10_i1------------
Well      i1
Species
RRHO
Geometry[angstrom]   20
C  -0.148091887   0.0667582179   0.0862486347
C  -0.0650659088   0.0525006918   1.4269282544
C  1.3433782457   0.0193898412   1.8329431884
C  2.1338586123   0.0116999981   0.6661694174
C  1.2268163446   0.0446139993   -0.5492213045
C  3.5168088264   -0.0210569114   0.7546997831
C  4.1142296668   -0.0462527784   2.0198278636
C  3.3316891988   -0.0362149898   3.1752959479
C  1.9386917056   -0.0029184267   3.0927248568
C  1.4363915606   -1.1221573368   -1.5315083074
H  4.1346204606   -0.0263288581   -0.1376558262
H  3.8120810089   -0.054898175   4.147362504
H  1.3345926949   0.0053482231   3.9937796274
H  -0.8991736513   0.0649976385   2.1176263531
H  1.2944694919   -2.0802185036   -1.0258149066
H  2.4455962255   -1.1037443716   -1.9506716324
H  0.7274201392   -1.0633232901   -2.3619297055
H  -1.0611025561   0.0893026785   -0.4955377858
H  5.1949467987   -0.0732246806   2.1036174262
H  1.3847010226   0.9856870338   -1.0962333887
Core  RigidRotor
SymmetryFactor  1.0
End
Frequencies[1/cm]  54
112.3226        164.2919        239.9941
274.1416        289.1277        410.1819
442.1065        526.5282        553.0530
576.4507        619.8479        726.3766
741.3991        750.6582        776.8943
808.1832        868.6441        882.2438
912.7021        943.7287        963.0817
986.3983        1011.8630       1041.9247
1074.9259       1089.3478       1098.8637
1135.4114       1177.0162       1187.9488
1223.0780       1250.6298       1300.5135
1312.5262       1344.8023       1390.5183
1408.6208       1486.9040       1489.5018
1499.6752       1499.9804       1603.1318
1636.3012       1649.5572       2989.3338
```

3028.0415 3091.5126 3101.3863
3155.3033 3161.8663 3172.9660
3184.8260 3185.8501 3207.6164
ZeroEnergy[kcal/mol] -68.6
ElectronicLevels[1/cm] 1
0 1
End
End
!---------------------------------
!-------c10h10_i2------------
Well i2
Species
RRHO
Geometry[angstrom] 20
C -0.0029011396 0.0387383396 -0.0514920168
C -0.0831183421 -0.0061435877 1.3872775983
C 1.1712104868 -0.0336275838 2.1768051579
C 2.434793676 -0.0146569557 1.4828969973
C 2.4441410689 0.0252537098 0.1272961815
C 1.2161746397 0.0521479611 -0.6456384411
C 0.8596373362 -0.0757105014 3.4986887541
C -0.6365138627 -0.0578236727 3.6562317309
C -1.141322093 -0.0318382921 2.2392078172
C -1.2081269174 -1.1959606663 4.5247723727
H 3.3603554196 -0.033022419 2.0482276775
H 1.2926904292 0.0849084928 -1.7270346598
H -0.9128474783 0.0605743901 -0.6415103406
H -2.1904678234 -0.0243953027 1.9741915454
H -0.9581680662 -2.1679611567 4.0938880027
H -2.296261798 -1.1203622387 4.595777996
H -0.7997410939 -1.1530960908 5.5377758584
H -0.9144264772 0.8993012272 4.1312423978
H 3.386825105 0.0390479988 -0.4088962667
H 1.5519219304 -0.1063746517 4.3298306372
Core RigidRotor
SymmetryFactor 1.0
End
Frequencies[1/cm] 54
95.8576 174.0117 233.4008
256.2487 299.5536 427.2551
450.6676 491.6671 528.2161
572.9764 583.0614 688.3405
695.9362 738.8543 774.8988
799.4304 838.2060 853.6435
894.8421 905.4379 965.7227
977.6229 978.2699 998.0150
998.5364 1066.6489 1081.9745
1142.7516 1178.6989 1188.2818
1222.7763 1230.4504 1278.7435
1292.1686 1382.1872 1397.6532

1403.7517 1448.3535 1498.5167
1500.4552 1546.9577 1579.2874
1614.7622 1687.0165 2956.4381
3029.1105 3092.6984 3103.8578
3156.6113 3163.9509 3176.2302
3185.3381 3202.1543 3204.7735
ZeroEnergy[kcal/mol] -47.1
ElectronicLevels[1/cm] 1
0 1
End
End
!----------------------------------
!-------c10h10_i3------------
Well i3
Species
RRHO
Geometry[angstrom] 20
C -0.4632682478 0.3020180051 -1.9318365062
C -0.4711089164 0.0677434491 -0.5578767494
C 0.7373558429 0.0431915009 0.1677662476
C 1.9493041109 0.2508211165 -0.4703354525
C 1.9554412876 0.4859415471 -1.8500873458
C 0.7606152204 0.5106851948 -2.570300028
C 0.4179742609 -0.2296406865 1.6186219408
C -1.0937976815 -0.3545705548 1.6261758738
C -1.5696472161 -0.181141759 0.3773960214
C -1.8714905548 -0.6301547807 2.8727994971
H 2.882453676 0.2329128608 0.0838146057
H 0.7839222755 0.6943059229 -3.6389339256
H -1.3886175671 0.3225595008 -2.497720689
H -2.6152175677 -0.2185989517 0.094775994
H -1.5662427266 -1.5778332775 3.3317379214
H -2.9430649125 -0.6831971591 2.6700355104
H -1.7073585264 0.1497437076 3.6256024448
H 0.7572881495 0.5801833397 2.2772135677
H 2.8961287624 0.6502072554 -2.3636455813
H 0.8985083308 -1.1464642314 1.9836056529
Core RigidRotor
SymmetryFactor 1.0
End
Frequencies[1/cm] 54
119.5560 154.8879 211.0126
246.9788 296.4916 421.9985
427.1977 439.8495 475.8250
565.3915 607.8964 654.5523
733.0880 765.8561 801.3624
855.5672 870.5050 880.8529
891.2822 932.4126 949.7980
984.8501 1009.8937 1041.8848
1054.7357 1117.8454 1152.4559

1161.3682	1178.3921	1186.3216
1229.3506	1241.2137	1327.3254
1331.6634	1378.1210	1415.8969
1439.1447	1480.2436	1485.6977
1492.4716	1496.4890	1618.6210
1649.9346	1663.5757	3006.6459
3013.0829	3035.1512	3047.4373
3098.8962	3154.6076	3160.9527
3172.3901	3184.3748	3185.5952

ZeroEnergy[kcal/mol] -71.5
ElectronicLevels[1/cm] 1
0 1
End
End
!----------------------------------
!-------ch3_c9h7_p0------------
Bimolecular p0
Fragment c9h7
RRHO
Geometry[angstrom] 16
C 6.7251E-6 0.0 -0.0067899103
C 5.405214E-4 0.0 1.3930385168
C 1.3782441902 0.0 1.8506967711
C 2.2062850245 0.0 0.6869028629
C 1.3227626622 0.0 -0.4647541128
C 3.5878449156 0.0 0.8095325846
C 4.1548633018 0.0 2.0950516977
C 3.3480782116 0.0 3.2291510301
C 1.9475808142 0.0 3.1154714187
H 1.6403910173 0.0 -1.4986755909
H 4.2261134274 0.0 -0.0678746418
H 3.8046257482 0.0 4.2124022047
H 1.3284188847 0.0 4.0064725223
H -0.8724838361 0.0 2.0319019107
H -0.8804382002 0.0 -0.6335630148
H 5.2334485921 0.0 2.203874751
Core RigidRotor
SymmetryFactor 1
End
Frequencies[1/cm] 42

199.1610	240.5161	391.8290
414.1648	534.9722	549.1649
560.3268	578.8571	713.0492
744.4367	754.1336	760.5516
804.5421	864.4476	874.9078
887.3348	902.2059	943.0014
979.8202	1013.5489	1034.4682
1081.1772	1092.5515	1181.2233
1185.1137	1212.2337	1219.4915
1329.3270	1371.6508	1384.5319

1463.7594 1488.4738 1489.2815
1617.7974 1625.1073 3159.9688
3165.4443 3175.9796 3189.2696
3203.5608 3211.0357 3228.6412
ZeroEnergy[kcal/mol] 0.0
ElectronicLevels[1/cm] 1
0 2
End
Fragment ch3
RRHO
Geometry[angstrom] 4
C 0.0 1.995E-7 0.0
H 0.0 1.0804553795 0.0
H 0.9357017515 -0.5402277895 0.0
H -0.9357017515 -0.5402277895 0.0
Core RigidRotor
SymmetryFactor 6
End
Frequencies[1/cm] 6
505.1501 1403.0831 1403.0885
3103.6402 3282.6573 3282.6683
ZeroEnergy[kcal/mol] 0.0
ElectronicLevels[1/cm] 1
0 2
End
GroundEnergy[kcal/mol] 0.0
End
!-------h_c10h9_p1------------
Bimolecular p1
Fragment c10h9
RRHO
Geometry[angstrom] 19
C 0.400357 0.954177 0.0
C -0.16028 -0.358173 0.0
C 0.666652 -1.472953 0.0
C 2.057725 -1.285784 0.0
C 2.604179 -0.004322 0.0
C 1.777006 1.129251 0.0
C -0.708489 1.890534 0.0
C -1.612436 -0.21536 0.0
C -2.593341 -1.337872 0.0
C -1.898651 1.161044 0.0
H 0.254452 -2.476725 0.0
H 3.681307 0.119062 0.0
H 2.21253 2.123037 0.0
H -0.61916 2.968561 0.0
H -2.461241 -1.9801685 0.878959
H -2.462241 -1.9801685 -0.878959
H -3.621311 -0.971083 0.0
H -2.894708 1.582929 0.0

H 2.714047   -2.148703   0.0
Core RigidRotor
SymmetryFactor 1
End
Frequencies[1/cm] 51

102.2975	142.0659	205.5237
229.3953	308.2155	416.4227
459.6878	524.2789	556.6522
558.2369	597.5389	692.5842
724.0869	756.5346	757.9911
779.1648	864.1946	871.3037
886.9060	940.5644	957.4496
978.0569	1030.3384	1034.4476
1043.1175	1085.4469	1104.2817
1180.0009	1190.6339	1214.3339
1285.5876	1322.7418	1369.1508
1405.1409	1433.5558	1441.5595
1475.3238	1484.7390	1491.1617
1507.4846	1618.0643	1624.1122
3005.3032	3043.5726	3103.3542
3158.2164	3163.9754	3174.4908
3187.6888	3200.8656	3218.5419

ZeroEnergy[kcal/mol]   0.0
ElectronicLevels[1/cm]   1
0 2
End
Fragment   H
Atom
Mass[amu]   1
ElectronicLevels[1/cm]   1
0   2
End
GroundEnergy[kcal/mol] 7.1
End
!-------h_c10h9_p2------------
Bimolecular   p2
Fragment   c10h9
RRHO
Geometry[angstrom]   19
C 1.1640393806   1.1348827835   -1.065889E-4
C -0.2270401106   0.7140866126   -8.57467E-5
C -0.2284991681   -0.7140509708   9.01415E-5
C 1.1567908259   -1.1369662868   1.700585E-4
C 1.9854471733   0.0032929323   4.20454E-5
C -1.4259456412   -1.4141254205   1.668385E-4
C -2.6334792152   -0.6941729947   6.1729E-5
C -2.6314098853   0.6964669294   -1.120873E-4
C -1.4215775428   1.4150716772   -1.849803E-4
C 3.4876041184   -0.001835191   -1.7457E-6
H -1.4382409944   -2.4991244486   3.044896E-4

H -3.5723598131 1.2349272034 -1.910518E-4
H -1.432977907 2.5000710984 -3.181619E-4
H 1.5075542035 2.1616769483 -2.069657E-4
H 3.8845717017 1.015395051 0.0012423636
H -3.5758044988 -1.2301182283 1.179947E-4
H 1.4976686195 -2.16482257 3.186176E-4
H 3.8869144233 -0.5156717345 0.8802608873
H 3.8868833303 -0.513475391 -0.8815678373
Core RigidRotor
SymmetryFactor 1
End
Rotor Hindered ! CH3
Group 15 18 19
Axis 5 10
Symmetry 3
Potential[kcal/mol] 4
0.0 0.019 0.013 0.025
End
Frequencies[1/cm] 50

126.9905	239.2772	
248.0202	309.2232	420.2691
446.7713	484.7658	537.9351
560.5027	573.8546	654.2678
740.8150	742.8119	787.5784
798.7529	823.9302	870.9552
886.0648	939.5100	960.0946
974.6346	976.1699	1033.3524
1055.5575	1088.4750	1154.3733
1171.3032	1181.7303	1212.3972
1219.8208	1288.6825	1337.6311
1400.0241	1423.8380	1482.1685
1486.9546	1488.5235	1497.2997
1537.1147	1617.3793	1627.6591
3020.8336	3066.4202	3099.1409
3158.8814	3164.2822	3174.9782
3188.5274	3196.9769	3200.0597

ZeroEnergy[kcal/mol] 0.0
ElectronicLevels[1/cm] 1
0 2
End
Fragment H
Atom
Mass[amu] 1
ElectronicLevels[1/cm] 1
0 2
End
GroundEnergy[kcal/mol] 9.7
End
!-------barrier_ts3------------
Barrier ts3 i1 i2

RRHO
Geometry[angstrom] 20
C 0.0601426018 -0.0801791298 -0.0296681755
C 0.0720828017 -0.0254254241 1.3781515637
C 1.4355482756 -0.1319901587 1.8473238917
C 2.2675170695 -0.2452631126 0.7142667705
C 1.3983743697 -0.1940167886 -0.4646650116
C 1.7292119276 -0.1017950007 3.2299576803
C 0.6956822834 0.0161562086 4.1286403707
C -0.6483333893 0.1289577193 3.6741395504
C -0.9659907319 0.1187704837 2.336906266
C 2.2945536109 1.4959947117 -0.3128252407
H 2.7556722952 -0.1832811029 3.5735838069
H -1.4397637081 0.2275056719 4.4099889044
H -1.997781815 0.2114501073 2.0148020184
H -0.8060522328 -0.0168565004 -0.671257961
H 1.6821432706 2.0930814669 0.3522130492
H 2.09818448 1.7311104031 -1.3574840281
H 3.3599649302 1.6096808281 -0.1191237784
H 1.7086902747 -0.5240959113 -1.4463657779
H 0.8968358266 0.0257541087 5.1937415172
H 3.3101048596 -0.5249005802 0.7020805839
Core RigidRotor
SymmetryFactor 1.0
End
Tunneling Eckart
ImaginaryFrequency[1/cm] 748.0434
WellDepth[kcal/mol] 54.6
WellDepth[kcal/mol] 33.1
End
Frequencies[1/cm] 53

135.8540	188.8372	
223.3598	306.1774	379.9923
431.4193	473.0773	505.5166
556.3826	580.8540	599.6464
703.3276	753.3344	754.2485
780.8846	827.9242	858.1041
877.7790	885.8266	898.1922
950.6810	958.2176	985.9852
1015.5480	1019.3999	1057.2230
1079.2300	1146.2517	1174.8088
1209.5403	1243.8566	1259.4576
1288.3791	1363.8022	1376.4343
1388.6598	1418.1441	1442.8708
1481.3705	1488.7333	1534.9982
1569.9442	1653.8833	3054.1838
3131.1237	3152.0371	3157.5219
3171.3199	3183.8851	3201.7358
3211.1448	3224.6253	3231.1608

ZeroEnergy[kcal/mol] -14.0

ElectronicLevels[1/cm] 1
0 1
End
!----------------------------------
!-------barrier_ts5------------
Barrier ts5 i2 i3
RRHO
Geometry[angstrom] 20
C 0.0499046104 0.0667334723 -0.0771857799
C 1.21426E-5 -0.1604125044 1.3261097654
C 1.2343138456 -0.1773510232 2.0763989669
C 2.4733632511 0.0282637916 1.4202006588
C 2.4819899108 0.2180135445 0.0610031078
C 1.2675241029 0.2395723584 -0.6844718469
C 0.9171509219 -0.388829472 3.4311817745
C -0.5702894234 -0.4704824672 3.5274421778
C -1.0768338561 -0.3438427298 2.2058081858
C -1.3110715529 -0.9986194253 4.728348102
H 3.3996022178 0.0195429257 1.9852659952
H 1.3160479334 0.4001137098 -1.7562140185
H -0.8666351805 0.0919989383 -0.6570045071
H -2.1261111103 -0.3572600097 1.9469467925
H -1.3319466616 -2.0920684572 4.7068913456
H -2.3430669685 -0.6413305162 4.7430147439
H -0.8344840416 -0.6947703084 5.6638762398
H -0.0164849055 0.6141082942 3.7870762519
H 3.4220354001 0.3580256798 -0.4608732763
H 1.5828643638 -0.5184438013 4.2723873208
Core RigidRotor
SymmetryFactor 1.0
End
Tunneling Eckart
ImaginaryFrequency[1/cm] 1173.0932
WellDepth[kcal/mol] 15.4
WellDepth[kcal/mol] 39.8
End
Frequencies[1/cm] 53

131.5075	173.2842	
228.9907	260.9813	311.0955
440.0861	453.4274	492.8856
563.7043	600.1463	629.6141
665.4268	719.5628	746.2410
788.0228	805.2946	831.9715
861.3245	906.6013	941.5641
956.3488	988.2586	1000.6119
1019.1173	1059.7231	1126.2366
1145.5834	1179.1462	1184.9479
1211.9745	1246.6876	1299.4794
1365.7524	1379.9064	1380.6985
1421.7726	1465.3107	1492.1948

```
1493.5279            1505.5064            1543.2281
1572.6042            1661.8275            2137.7041
3029.9319            3086.4130            3101.3521
3154.8319            3161.5373            3173.3436
3184.5163            3214.8331            3225.2599
ZeroEnergy[kcal/mol]  -31.7
ElectronicLevels[1/cm] 1
0  1
End
!----------------------------------
 Barrier     ts1   i1  p0 #
    RRHO
      Stoichiometry C10H10
      Core     Rotd
 File ts1_flux.out
 SymmetryFactor 1.0
     End

Frequencies[1/cm]  48
199.1610             240.5161             391.8290
414.1648             534.9722             549.1649
560.3268             578.8571             713.0492
744.4367             754.1336             760.5516
804.5421             864.4476             874.9078
887.3348             902.2059             943.0014
979.8202            1013.5489            1034.4682
1081.1772            1092.5515            1181.2233
1185.1137            1212.2337            1219.4915
1329.3270            1371.6508            1384.5319
1463.7594            1488.4738            1489.2815
1617.7974            1625.1073            3159.9688
3165.4443            3175.9796            3189.2696
3203.5608            3211.0357            3228.6412
505.1501            1403.0831            1403.0885
3103.6402            3282.6573            3282.6683
ZeroEnergy[kcal/mol]      0.0
ElectronicLevels[1/cm]        1
0  1
End
!-----------------------------------------------------------
 Barrier     ts2   i1  p1 #
    RRHO
      Stoichiometry C10H10
      Core     Rotd
 File ts2_flux.out
 SymmetryFactor 1.0
     End

Frequencies[1/cm]  51
102.2975             142.0659             205.5237
```

229.3953	308.2155	416.4227
459.6878	524.2789	556.6522
558.2369	597.5389	692.5842
724.0869	756.5346	757.9911
779.1648	864.1946	871.3037
886.9060	940.5644	957.4496
978.0569	1030.3384	1034.4476
1043.1175	1085.4469	1104.2817
1180.0009	1190.6339	1214.3339
1285.5876	1322.7418	1369.1508
1405.1409	1433.5558	1441.5595
1475.3238	1484.7390	1491.1617
1507.4846	1618.0643	1624.1122
3005.3032	3043.5726	3103.3542
3158.2164	3163.9754	3174.4908
3187.6888	3200.8656	3218.5419

ZeroEnergy[kcal/mol] 7.1
ElectronicLevels[1/cm] 1
0 1
End
!---
Barrier ts7 i2 p0 #
RRHO
Stoichiometry C10H10
Core Rotd
File ts7_flux.out
SymmetryFactor 1.0
End

Frequencies[1/cm] 48

199.1610	240.5161	391.8290
414.1648	534.9722	549.1649
560.3268	578.8571	713.0492
744.4367	754.1336	760.5516
804.5421	864.4476	874.9078
887.3348	902.2059	943.0014
979.8202	1013.5489	1034.4682
1081.1772	1092.5515	1181.2233
1185.1137	1212.2337	1219.4915
1329.3270	1371.6508	1384.5319
1463.7594	1488.4738	1489.2815
1617.7974	1625.1073	3159.9688
3165.4443	3175.9796	3189.2696
3203.5608	3211.0357	3228.6412
505.1501	1403.0831	1403.0885
3103.6402	3282.6573	3282.6683

ZeroEnergy[kcal/mol] 0.0
ElectronicLevels[1/cm] 1
0 1
End

!--
Barrier ts4 i2 p2 #

Geometry[angstrom] 20
C 1.1640393806 1.1348827835 -1.065889E-4
C -0.2270401106 0.7140866126 -8.57467E-5
C -0.2284991681 -0.7140509708 9.01415E-5
C 1.1567908259 -1.1369662868 1.700585E-4
C 1.9854471733 0.0032929323 4.20454E-5
C -1.4259456412 -1.4141254205 1.668385E-4
C -2.6334792152 -0.6941729947 6.1729E-5
C -2.6314098853 0.6964669294 -1.120873E-4
C -1.4215775428 1.4150716772 -1.849803E-4
C 3.4876041184 -0.001835191 -1.7457E-6
H -1.4382409944 -2.4991244486 3.044896E-4
H -3.5723598131 1.2349272034 -1.910518E-4
H -1.432977907 2.5000710984 -3.181619E-4
H 1.5075542035 2.1616769483 -2.069657E-4
H 3.8845717017 1.015395051 0.0012423636
H -3.5758044988 -1.2301182283 1.179947E-4
H 1.4976686195 -2.16482257 3.186176E-4
H 3.8869144233 -0.5156717345 0.8802608873
H 3.8868833303 -0.513475391 -0.8815678373
H 20.0000000 0.0000000 0.0000000

RRHO
Stoichiometry C10H10
Core Rotd
File ts4_flux.out
SymmetryFactor 1.0
End

Rotor Hindered ! CH3
Group 15 18 19
Axis 5 10
Symmetry 3
Potential[kcal/mol] 4
0.0 0.019 0.013 0.025
End

Frequencies[1/cm] 50
126.9905 239.2772
248.0202 309.2232 420.2691
446.7713 484.7658 537.9351
560.5027 573.8546 654.2678
740.8150 742.8119 787.5784
798.7529 823.9302 870.9552
886.0648 939.5100 960.0946
974.6346 976.1699 1033.3524
1055.5575 1088.4750 1154.3733

```
1171.3032             1181.7303             1212.3972
1219.8208             1288.6825             1337.6311
1400.0241             1423.8380             1482.1685
1486.9546             1488.5235             1497.2997
1537.1147             1617.3793             1627.6591
3020.8336             3066.4202             3099.1409
3158.8814             3164.2822             3174.9782
3188.5274             3196.9769             3200.0597
ZeroEnergy[kcal/mol]      9.7
ElectronicLevels[1/cm]       1
0  1
End
!----------------------------------------------------------
 Barrier     ts6   i3  p2 #

RRHO
      Stoichiometry C10H10
      Core     Rotd
 File ts6_flux.out
 SymmetryFactor 1.0
 End

Frequencies[1/cm]  51
35.9                 126.9905             239.2772
248.0202             309.2232             420.2691
446.7713             484.7658             537.9351
560.5027             573.8546             654.2678
740.8150             742.8119             787.5784
798.7529             823.9302             870.9552
886.0648             939.5100             960.0946
974.6346             976.1699             1033.3524
1055.5575            1088.4750            1154.3733
1171.3032            1181.7303            1212.3972
1219.8208            1288.6825            1337.6311
1400.0241            1423.8380            1482.1685
1486.9546            1488.5235            1497.2997
1537.1147            1617.3793            1627.6591
3020.8336            3066.4202            3099.1409
3158.8814            3164.2822            3174.9782
3188.5274            3196.9769            3200.0597
ZeroEnergy[kcal/mol]      9.7
ElectronicLevels[1/cm]       1
0  1
End
!===========================================================
End
```

Supplementary References

1 Zhao, L. *et al.* Reactivity of the indenyl radical (C_9H_7) with acetylene (C_2H_2) and vinylacetylene (C_4H_4). *ChemPhysChem* **20,** 1437-1447 (2019).

2 Lide, D. R. Ionization potentials of atoms and atomic ions. *Handbook of chemistry and physics***,** 10-211 (1992).

3 Colket, M. B. & Seery, D. J. Reaction mechanisms for toluene pyrolysis. *Proc. Combust. Inst.* **25,** 883-891 (1994).

4 Jin, H. *et al.* An experimental study of indene pyrolysis with synchrotron vacuum ultraviolet photoionization mass spectrometry. *Phys. Chem. Chem. Phys.* **21,** 5510-5520 (2019).
